# Supplementary material for: Constitutively active RAS prolongs Cdc42 signalling, while MAPK signalling is attenuated during fission yeast mating
Source: PLoS Genet. 2026 Apr 17;22(4):e1012117. doi: 10.1371/journal.pgen.1012117 (PMC13108886; doi:10.1371/journal.pgen.1012117)
Supplement: S2 Table — Strains used in this study. (DOCX) [file pgen.1012117.s024.docx]

**Supplementary Table S2 : Strains used in this study**

**Figure 1**

**C, D**

**KT3082** *h^90^ ade6.M216 leu1.32 spk1-GFP-2xFLAG::Kan^R^*

**E, F**

**KT3435** *h^90^ ade6-M216 leu1.32 byr1.DD::Hyg^R^ spk1-GFP-2xFLAG::Kan^R^*

**G, H**

**KT3084** *h^90^ ade6.M210 leu1.32 ras1.G17V::LEU2^+^ spk1-GFP-2xFLAG::Kan^R^*

**Figure 2**

**A, B**

**KT3439** *h^90^ ade6.M210 leu1.32 ras1.G17V::LEU2^+^ byr1.DD::Hyg^R^ spk1-GFP-2xFLAG::Kan^R^*

**C**

**KT4300** *h^90^ ade6.M216 leu1.32 byr1::ClonNAT^R^ spk1-GFP-2xFLAG::Kan^R^*

**KT5215** *h^90^ ade6.M210 leu1.32 ras1.G17V::LEU2^+^ byr1::ClonNAT^R^ spk1-GFP-2xFLAG::Kan^R^*

**Figure 3**

**A**

**KT3082** *h^90^ ade6.M216 leu1.32 spk1-GFP-2xFLAG::Kan^R^*

**KT4061** *h^90^ ade6.M216 leu1.32 scd1::ClonNAT^R^ spk1-GFP-2xFLAG::Kan^R^*

**KT4056** *h^90^ ade6.M210 leu1.32 ras1.G17V::LEU2^+^ scd1::ClonNAT^R^ spk1-GFP-2xFLAG::Kan^R^*

**B**

**KT3082** *h^90^ ade6.M216 leu1.32 spk1-GFP-2xFLAG::Kan^R^*

**KT4061** *h^90^ ade6.M216 leu1.32 scd1::ClonNAT^R^ spk1-GFP-2xFLAG::Kan^R^*

**C**

**KT4047** *h^90^ ade6.M216 leu1.32 byr1.DD::Hyg^R^ scd1::ClonNAT^R^ spk1-GFP-2xFLAG::Kan^R^*

**D**

**KT4061** *h^90^ ade6.M216 leu1.32 scd1::ClonNAT^R^ spk1-GFP-2xFLAG::Kan^R^*

**KT4047** *h^90^ ade6.M216 leu1.32 byr1.DD::Hyg^R^ scd1::ClonNAT^R^ spk1-GFP-2xFLAG::Kan^R^*

**KT4056** *h^90^ ade6.M210 leu1.32 ras1.G17V::LEU2^+^ scd1::ClonNAT^R^ spk1-GFP-2xFLAG::Kan^R^*

**E**

**KT4323** *h^90^ ade6.M216 leu1.32 ras1::ClonNAT^R^ spk1-GFP-2xFLAG::Kan^R^*

**KT3435** *h^90^ ade6-M216 leu1.32 byr1.DD::Hyg^R^ spk1-GFP-2xFLAG::Kan^R^*

**KT4359** *h^90^ ade6.M216 leu1.32 byr1.DD::Hyg^R^ ras1::ClonNAT^R^ spk1-GFP-2xFLAG::Kan^R^*

**F**

**KT3763** *h^90^ ade6.M216 leu1.32 byr2::ClonNAT^R^ spk1-GFP-2xFLAG::Kan^R^*

**KT3435** *h^90^ ade6-M216 leu1.32 byr1.DD::Hyg^R^ spk1-GFP-2xFLAG::Kan^R^*

**KT4010** *h^90^ ade6-M216 leu1.32 byr1.DD::Hyg^R^ byr2::ClonNAT^R^ spk1-GFP-2xFLAG::Kan^R^*

**G**

**KT4323** *h^90^ ade6.M216 leu1.32 ras1::ClonNAT^R^ spk1-GFP-2xFLAG::Kan^R^*

**KT4359** *h^90^ ade6.M216 leu1.32 byr1.DD::Hyg^R^ ras1::ClonNAT^R^ spk1-GFP-2xFLAG::Kan^R^*

**KT3763** *h^90^ ade6.M216 leu1.32 byr2::ClonNAT^R^ spk1-GFP-2xFLAG::Kan^R^*

**KT4010** *h^90^ ade6-M216 leu1.32 byr1.DD::Hyg^R^ byr2::ClonNAT^R^ spk1-GFP-2xFLAG::Kan^R^*

**Fig. 4**

**A, B**

**KT5077** *h^90^ ade6.M210 leu1.32 ura4.294::[Pshk1:ScGIC2 CRIB:GFP3:ura4^+^]*

**KT5082** *h^90^ ade6.M216 leu1.32 ras1.G17V::LEU2^+^ ura4.294::[Pshk1:ScGIC2 CRIB:GFP3:ura4^+^]*

**Fig. 5**

**A**

**KT5940** *h^90^ ade6.M216 leu1.32 ras1.G17V::Hyg^R^ spk1-GFP-2xFLAG::Kan^R^*

**B**

**KT5938** *h^90^ ade6.M216 leu1.32 ras1.G17V::Hyg^R^ ura4.294::[Pshk1:ScGIC2 CRIB:GFP3:ura4^+^]*

**Fig. 6**

**A, B**

**KT4376** *h^90^ ade6.M216 leu1.32 ste4::ClonNAT^R^ spk1-GFP-2xFLAG::Kan^R^*

**KT5143** *h^90^ ade6.M210 leu1.32 ste4::ClonNAT^R^ ras1.G17V::LEU2^+^ spk1-GFP-2xFLAG::Kan^R^*

**KT5136** *h^90^ ade6-M216 leu1.32 ste4::ClonNAT^R^ byr1.DD::Hyg^R^ spk1-GFP-2xFLAG::Kan^R^*

**C, D**

**KT4333** *h^90^ ade6.M216 leu1.32 ste6::ClonNAT^R^ spk1-GFP-2xFLAG::Kan^R^*

**KT4998** *h^90^ ade6.M210 leu1.32 ste6::Hyg^R^ ras1.G17V::LEU2^+^ spk1-GFP-2xFLAG::Kan^R^*

**KT5139** *h^90^ ade6-M216 leu1.32 ste6::ClonNAT^R^ byr1.DD::Hyg^R^ spk1-GFP-2xFLAG::Kan^R^*

**Fig. 7**

**A, B**

**KT4335** *h^90^ ade6.M216 leu1.32 gpa1::ClonNAT^R^ spk1-GFP-2xFLAG::Kan^R^*

**KT5023** *h^90^ ade6.M210 leu1.32 gpa1::ClonNAT^R^ ras1.G17V::LEU2^+^ spk1-GFP-2xFLAG::Kan^R^*

**KT4353** *h^90^ ade6-M216 leu1.32 gpa1::ClonNAT^R^ byr1.DD::Hyg^R^ spk1-GFP-2xFLAG::Kan^R^*

**KT5035** *h^90^ ade6.M210 leu1.32 gpa1::ClonNAT^R^ ras1.G17V::LEU2^+^ byr1.DD::Hyg^R^ spk1-GFP-2xFLAG::Kan^R^*

**C, D**

**KT4190** *h^-^ ade6.M216 leu1.32 spk1-GFP-2xFLAG::Kan^R^*

**KT5059** *h^-^ ade6.M216 leu1.32 ura4.d18 gpa1.QL::ura4^+^ spk1-GFP-2xFLAG::Kan^R^*

**KT4233** *h^-^ ade6.M216 leu1.32 ras1.G17V::LEU2^+^ spk1-GFP-2xFLAG::Kan^R^*

**KT5070** *h^-^ ade6.M216 leu1.32 ura4.d18 ras1::ClonNAT^R^ gpa1.QL::ura4^+^ spk1-GFP-2xFLAG::Kan^R^*

**KT4194** *h^-^ ade6.M216 leu1.32 byr1.DD::Hyg^R^ spk1-GFP-2xFLAG::Kan^R^*

**Supplementary Figure S1**

**D, E and F**

**KT301** *h^90^ ade6-M216 leu1.32*

**KT3082** *h^90^ ade6.M216 leu1.32 spk1-GFP-2xFLAG::Kan^R^*

**G**

**KT3082** *h^90^ ade6.M216 leu1.32 spk1-GFP-2xFLAG::Kan^R^*

**KT4300** *h^90^ ade6.M216 leu1.32 byr1::ClonNAT^R^ spk1-GFP-2xFLAG::Kan^R^*

**Supplementary Figure S2 and S3**

**KT3082** *h^90^ ade6.M216 leu1.32 spk1-GFP-2xFLAG::Kan^R^*

**KT3435** *h^90^ ade6-M216 leu1.32 byr1.DD::Hyg^R^ spk1-GFP-2xFLAG::Kan^R^*

**KT3084** *h^90^ ade6.M210 leu1.32 ras1.G17V::LEU2^+^ spk1-GFP-2xFLAG::Kan^R^*

**KT3439** *h^90^ ade6.M210 leu1.32 ras1.G17V::LEU2^+^ byr1.DD::Hyg^R^ spk1-GFP-2xFLAG::Kan^R^*

**KT4061** *h^90^ ade6.M216 leu1.32 scd1::ClonNAT^R^ spk1-GFP-2xFLAG::Kan^R^*

**Supplementary Figure S4 and S5**

**A and B**

**KT5951** *h^90^ ade6.M216 leu1 spk1-GFP-2xFLAG::Kan^R^ smd2-tdTomato::Hyg^R^*

**Supplementary Figure S6**

**KT3082** *h^90^ ade6.M216 leu1.32 spk1-GFP-2xFLAG::Kan^R^*

**KT3982** *h^90^ ade6.M210 leu1.32 fus1::Hyg^R^ spk1-GFP-2xFLAG::Kan^R^*

**Supplementary Figure S7**

**A and B**

**KT3435** *h^90^ ade6-M216 leu1.32 byr1.DD::Hyg^R^ spk1-GFP-2xFLAG::Kan^R^*

**Supplementary Figure S8**

**A and D**

**KT3435** *h^90^ ade6-M216 leu1.32 byr1.DD::Hyg^R^ spk1-GFP-2xFLAG::Kan^R^*

**B and D**

**KT3084** *h^90^ ade6.M210 leu1.32 ras1.G17V::LEU2^+^ spk1-GFP-2xFLAG::Kan^R^*

**Supplementary Figure S9**

**KT5107** *h^90^ ade6.M210 leu1.32 ras1::ClonNAT^R^ ura4.294::[Pshk1:ScGIC2 CRIB:GFP3:ura4^+^]*

**KT5077** *h^90^ ade6.M210 leu1.32 ura4.294::[Pshk1:ScGIC2 CRIB:GFP3:ura4^+^]*

**KT5082** *h^90^ ade6.M216 leu1.32 ras1.G17V::LEU2^+^ ura4.294::[Pshk1:ScGIC2 CRIB:GFP3:ura4^+^]*

**KT5551** *h^90^ ade6.M210 leu1.32 rga4::Hyg^R^ ura4.294::[Pshk1:ScGIC2 CRIB:GFP3:ura4^+^]*

**KT5554** *h^90^ ade6.M216 leu1.32 ras1.G17V::LEU2^+^ rga4::Hyg^R^ ura4.294::[Pshk1:ScGIC2 CRIB:GFP3:ura4^+^]*

**Supplementary Figure S10**

**A and D**

**KT4061** *h^90^ ade6.M216 leu1.32 scd1::ClonNAT^R^ spk1-GFP-2xFLAG::Kan^R^*

**KT4056** *h^90^ ade6.M210 leu1.32 ras1.G17V::LEU2^+^ scd1::ClonNAT^R^ spk1-GFP-2xFLAG::Kan^R^*

**KT4047** *h^90^ ade6.M216 leu1.32 byr1.DD::Hyg^R^ scd1::ClonNAT^R^ spk1-GFP-2xFLAG::Kan^R^*

**B and E**

**KT4323** *h^90^ ade6.M216 leu1.32 ras1::ClonNAT^R^ spk1-GFP-2xFLAG::Kan^R^*

**KT3435** *h^90^ ade6-M216 leu1.32 byr1.DD::Hyg^R^ spk1-GFP-2xFLAG::Kan^R^*

**KT4359** *h^90^ ade6.M216 leu1.32 byr1.DD::Hyg^R^ ras1::ClonNAT^R^ spk1-GFP-2xFLAG::Kan^R^*

**C and F**

**KT3763** *h^90^ ade6.M216 leu1.32 byr2::ClonNAT^R^ spk1-GFP-2xFLAG::Kan^R^*

**KT3435** *h^90^ ade6-M216 leu1.32 byr1.DD::Hyg^R^ spk1-GFP-2xFLAG::Kan^R^*

**KT4010** *h^90^ ade6-M216 leu1.32 byr1.DD::Hyg^R^ byr2::ClonNAT^R^ spk1-GFP-2xFLAG::Kan^R^*

**Supplementary Figure S11**

**KT5940** *h^90^ ade6.M216 leu1.32 ras1.G17V::Hyg^R^ spk1-GFP-2xFLAG::Kan^R^*

**Supplementary Figure S12**

**KT5938** *h^90^ ade6.M216 leu1.32 ras1.G17V::Hyg^R^ ura4.294::[Pshk1:ScGIC2 CRIB:GFP3:ura4^+^]*

**Supplementary Figure S13**

**A**

**KT5032** *h^90^ ade6.M210 leu1.32 byr2-GFP::Kan^R^*

**B and C**

**KT4382** *h^90^ ade6-M216 leu1 scd1-GFP-2xFLAG::Kan^R^*

**KT5135** *h^90^ ade6.M210 leu1.32 ura4.d18* *GFP-byr2+*

**Supplementary Figure S14**

**A and C**

**KT4376** *h^90^ ade6.M216 leu1.32 ste4::ClonNAT^R^ spk1-GFP-2xFLAG::Kan^R^*

**KT5143** *h^90^ ade6.M210 leu1.32 ste4::ClonNAT^R^ ras1.G17V::LEU2^+^ spk1-GFP-2xFLAG::Kan^R^*

**KT5136** *h^90^ ade6-M216 leu1.32 ste4::ClonNAT^R^ byr1.DD::Hyg^R^ spk1-GFP-2xFLAG::Kan^R^*

**B and D**

**KT4333** *h^90^ ade6.M216 leu1.32 ste6::ClonNAT^R^ spk1-GFP-2xFLAG::Kan^R^*

**KT4998** *h^90^ ade6.M210 leu1.32 ste6::Hyg^R^ ras1.G17V::LEU2^+^ spk1-GFP-2xFLAG::Kan^R^*

**KT5139** *h^90^ ade6-M216 leu1.32 ste6::ClonNAT^R^ byr1.DD::Hyg^R^ spk1-GFP-2xFLAG::Kan^R^*

**Supplementary Figure S15**

**A**

**KT4335** *h^90^ ade6.M216 leu1.32 gpa1::ClonNAT^R^ spk1-GFP-2xFLAG::Kan^R^*

**KT5023** *h^90^ ade6.M210 leu1.32 gpa1::ClonNAT^R^ ras1.G17V::LEU2^+^ spk1-GFP-2xFLAG::Kan^R^*

**KT4353** *h^90^ ade6-M216 leu1.32 gpa1::ClonNAT^R^ byr1.DD::Hyg^R^ spk1-GFP-2xFLAG::Kan^R^*

**KT5035** *h^90^ ade6.M210 leu1.32 gpa1::ClonNAT^R^ ras1.G17V::LEU2^+^ byr1.DD::Hyg^R^ spk1-GFP-2xFLAG::Kan^R^*

**B**

**KT4190** *h^-^ ade6.M216 leu1.32 spk1-GFP-2xFLAG::Kan^R^*

**KT5059** *h^-^ ade6.M216 leu1.32 ura4.d18 gpa1.QL::ura4^+^ spk1-GFP-2xFLAG::Kan^R^*

**KT4233** *h^-^ ade6.M216 leu1.32 ras1.G17V::LEU2^+^ spk1-GFP-2xFLAG::Kan^R^*

**KT5070** *h^-^ ade6.M216 leu1.32 ura4.d18 ras1::ClonNAT^R^ gpa1.QL::ura4^+^ spk1-GFP-2xFLAG::Kan^R^*

**KT4194** *h^-^ ade6.M216 leu1.32 byr1.DD::Hyg^R^ spk1-GFP-2xFLAG::Kan^R^*

**Supplementary Figure S21**

**KT3084** *h^90^ ade6.M210 leu1.32 ras1.G17V::LEU2^+^ spk1-GFP-2xFLAG::Kan^R^*

**KT4037** *h^90^ ade6.M210 leu1.32 ras1.G17V::LEU2^+^ spk1-GFP-2xFLAG::Kan^R^ sxa2∆::clonNAT*

**Supplementary Figure S22**

**KT3435** *h^90^ ade6-M216 leu1.32 byr1.DD::Hyg^R^ spk1-GFP-2xFLAG::Kan^R^*

**KT4031** *h^90^ ade6-M216 leu1.32 byr1.DD::Hyg^R^ spk1-GFP-2xFLAG::Kan^R^ sxa2∆::clonNAT*
