# Supplementary material for: Constitutively active RAS prolongs Cdc42 signalling, while MAPK signalling is attenuated during fission yeast mating
Source: PLoS Genet. 2026 Apr 17;22(4):e1012117. doi: 10.1371/journal.pgen.1012117 (PMC13108886; doi:10.1371/journal.pgen.1012117)
Supplement: Attachment — Submitted filename: Response to the reviewers_2026.pdf [file pgen.1012117.s026.pdf]

Response to the Reviewers

We thank all the reviewers for their valuable and constructive comments and suggestions. Specific responses to each comment are provided below.

Reviewer 1

Major points

1. The authors establish a protocol to induce highly synchronous mating (Fig.1C), where the fraction of "mating" increased 0-80% from 7h-15h. However, mating results in fusion and diploid cells rapidly arrest mating, thus the fraction of actively mating cells is actually considerably lower than presented in the graph, which is misleading. Thus, reporting fused cells, cells that are shmooing and cells that are scanning and arrested (Bendezu and Martin, 2013) would be more informative. This, also raises the following questions:

1a. How much of the ppSpk1 drop can be explained by wildtype cells fusing? What is the profile in fus1Δ cells that never fuse?

Thank you very much for the helpful suggestion to improve the figure and for raising the important question.

We revised Fig. 1C accordingly. We also examined the time course ppSpk1 level in the *fus1Δ* mutant. The result, presented in Supplementary Fig. S6, demonstrated that the *fus1Δ* mutant retained a high ppSpk1 level, indicating that successful cell fusing is required for the downregulation of ppSpk1.

1b. Does the fact that WT cells fuse and numerous mutants fail to fuse complicate the comparisons between the mutants and controls at late timepoints in the experiment? This should be discussed where relevant.

Thank you very much for raising this important issue. We fully agree with the reviewer: comparing wildtype (mated) cells and sterile mutants at later timepoints is complex, and we need to be cautious in interpreting the data.

This work aims to understand the PS signalling that leads to successful mating. Hence, it was essential to examine mating-deficient mutants. Two sterile mutants, *byr1.DD* and

ras1.G17V, showed distinct phenotypes, which helped us to examine the PS signalling process without wildtype cells. They were also used as useful reference strains in the epistasis analyses of sterile mutants.

We included late timepoints in our study to confirm that longer incubation does not improve their sterile phenotypes. The generated data up to 24 hours was used in the mathematical modelling to “describe” our observation. However, the interpretation of ppSpk1 and CRIB-GFP readouts at later timepoints in both wildtype and sterile mutants requires careful consideration, as highlighted by the reviewer. The issue is discussed in the model building section as follows (L419):

We are aware that whilst the wildtype data represents the entire sexual differentiation process, including G1 arrest, pheromone sensing, mating, followed by DNA recombination and sporulation, all the sterile mutants are blocked at the stage before successfully completing mating. Therefore, we need to be cautious when interpreting differences in the later timepoint data. However, we still decided to include all time points in our attempt to “describe” our experimental results using a single signalling framework.

2. The authors highlight in the abstract that they implement the competition between the Ras1 effectors MAPKKKByr2 and Cdc42-GEFScd1. Two points regarding this are:
2a. The authors convincingly show that Byr2 and Scd1 compete to bind to Ras1-GTP in vitro. While these results show that Byr2 and Scd1 can compete, their protein levels relative to that Ras1-GTP will also be important (both Scd1 and Byr2 are low level proteins and Ras1 is abundant). The authors show in vivo that Ras1-binding domains of Byr2/Scd1 outcompete native proteins but this is done upon extreme overexpression of Byr2/Scd1 from the pREP1 vectors. How much competition of Byr2 and Scd1 there is at native levels thus remains unclear and should be explored further (e.g. changes in gene copy number)

Thank you very much for raising this critical issue. We are keen on addressing this point.

According to the data collated in PomBase, gene and protein expression levels of scd1, byr2 and spk1 are as follows;

	RNA level: G1 arrest (-N) Marguerat et al (2012)	Protein level G1 arrest (-N) Marguerat et al (2012)	RNA level: vegetative growth Marguerat et al (2012)	Protein level Vegetative growth Carpy et al (2014)
scd1	0.81	453	2.1	~500
byr2	0.3	unknown	0.88	unknown
spk1	0.89	unknown	0.59	~1200

From these numbers, the correlation between RNA and protein expression levels is not strong. Furthermore, whether the expressed proteins uniformly reside in the cell or exhibit specific cellular localisation patterns is unclear.

Therefore, we generated yeast strains harbouring endogenously GFP-tagged GFP-byr2 and scd1-GFP and conducted image analysis. Previous studies showed that a 3xGFP tag is preferable for higher sensitivity in detecting Scd1 than a 1xGFP tag. However, recent studies from the Das lab showed that Scd1-3xGFP and Scd1-mNG signals behave differently (https://doi.org/10.1101/2025.01.24.634833). Furthermore, the Byr2 function could be compromised with a tag; we previously generated cells expressing endogenously C-terminally GFP-tagged Byr2, which turned out to be sterile, even though the Byr2-GFP signal was very strong (Supplementary Fig. S13A). So, we went for the 1xGFP tag, and for Byr2, we GFP-tagged it at its N-terminal end.

Both Scd1-GFP and GFP-Byr2 signals were weak compared to the Spk1-GFP signal. In addition, both signals can quickly disappear when cells are placed under coverslips. We therefore could manage to detect GFP signals in only a few of these cells, which were undergoing mating: the newly forming zygotes showed enrichment of Scd1 and Byr2 at the fusing cell tips. The signal intensities of both proteins were comparable, but the GFP-Byr2 signal could potentially be a little stronger. Our observation supports the idea that these two molecules are available at the fusion site at comparable levels. The result is presented in Supplementary Figure S13. The matter clearly needs to be more rigorously addressed in the future, using GFP variants such as mNG or mStayGold, which produce higher-intensity signals.

2b. The authors do not discuss the importance of Byr2/Scd1 putative competition in the model nor do they use the model to explore its importance. Also, it is not clearly stated (nor immediately evident to this reviewer) how the model captures the proposed competition.

We thank the reviewer for pointing out an important inconsistency between the description of the model and its actual implementation. We agree that our original wording in the Discussion was misleading.

In the current mathematical model, we do not explicitly implement "competitive" binding between the two Ras1 effectors, Cdc42-GEF^{Scd1} and MAPKKK^{Byr2}. Instead, Ras1 activity is treated as a shared upstream input that independently regulates the Byr2–MAPK and Scd1–Cdc42 modules. The statement that the model "*incorporates assumptions that Byr2 and Scd1 compete for active Ras1*" was therefore misleading, and we have corrected this description in the revised manuscript as follows (L528): The model incorporates a delayed negative feedback path acting on the MAPK^{Spk1} axis, together with activation of the Byr1 node by the pheromone sensing unit (Ste4), Ras1 and the Cdc42 nodes.

The primary purpose of the model was to identify the mechanism responsible for the transient activation followed by attenuation of MAPK^{Spk1}. As shown by both experimental data and model fitting, this transient behavior is observed even in ras1.G17V cells, where Ras1 activity is constitutively high, and in scd1Δ cells, where competition between Byr2 and Scd1 cannot occur. These results indicate that Byr2–Scd1 competition is not required to explain the transient MAPK^{Spk1} dynamics addressed by the model. Instead, our analysis demonstrates that a delayed negative feedback acting on the MAPK^{Spk1} axis is sufficient to account for these dynamics. In the revised manuscript, we clarified the purpose of the modeling as follows (L527): We built a mathematical model to investigate the mechanism underlying the transient activation and subsequent attenuation of MAPK^{Spk1}.

We would like to note that Byr2–Scd1 competition may play important roles in other aspects of Ras1 signalling, such as pathway allocation or robustness. Furthermore, in a physiological context where the quantity of active Ras1 is finite, the Byr2–Scd1 competition

is likely to occur. While incorporating such competition into a mathematical framework represents a valuable future direction, doing so would substantially increase model complexity without contributing to the specific question addressed here. We also need to know the local molar stoichiometry of Ras1, Byr2, and Scd1, which is another important future task. Having considered these points, we have revised the Discussion to clarify both the scope and the limitations of the current model as follows (L581): In this study, we also uncovered experimental evidence that two Ras1 effectors, MAPKKKByr2 and Cdc42-GEFScd1, can compete for Ras1. While the physiological significance of this competition requires further investigation, it is likely to influence how Ras1 signalling is allocated between downstream pathways, thereby defining the physiological outcome. Although this competitive interaction was not explicitly addressed in the current mathematical model, extending the modelling framework to incorporate such competition will be an important future direction. Combined experimental and theoretical approaches may help clarify how Ras1 effector competition contributes to pathway robustness, signal prioritization, and cell fate decisions during mating.

In addition, we noticed during revision that part of the legend of Supplementary Figure S16 (previously Supplementary Fig. S11) incorrectly referred to “Model B” and should instead read “Model C”. This has been corrected in the revised manuscript.

3. The authors highlight in the abstract that they implement the pheromone signaling negative feedback and explain that this is a critical aspect for the model to capture the observed dynamics (Model B). They propose factors such as Sxa2 and Rgs1 as key regulators of negative feedback, but they do not experimentaly test their mutants regarding ppSpk1, tSpk1 nor CRIB, which would help stregthen the predictions of their model. In particular, the ppSpk1 delay in double mutants with byr1DD would be interesting.

We appreciate this valuable comment and measured the ppSpk1 and total Spk1 in the *by1.DD sxa2Δ* double mutant (shown in Supplementary Fig. S22) and *ras1.G17V sxa2Δ* double mutant (shown in Supplementary Fig. S21). Loss of Sxa2 is expected to increase the PS input.

Strikingly, the loss of *sxa2* increased the amplitude of the ppSpk1 activation in the *ras1.G17V* mutant (Fig. S21). The outcome supports our prediction that Sxa2 plays a role in the negative feedback that downregulates the ppSpk1 level.

Deletion of *sxa2* in the *byr1.DD* mutant cells did not accelerate the timing of ppSPK1 activation, indicating that the ppSpk1 activation timing is robustly defined by the expression level of *byr1.DD*, which may be mainly regulated by nitrogen-starvation (-N) signalling, but not the pheromone signalling.

Intriguingly, the *byr1.DD* morphology (the "paired" phenotype) was changed into the "elongated" phenotype. As the increase in local pheromone levels causes a loss of directional shmoo formation, we need to be cautious when interpreting this result. However, considering that the "elongated" phenotype is a representative morphology of *ras1.G17V*, where Cdc42 is hyperactivated, the *sxa2Δ*-induced compromised feedback circuit might have resulted in the enhanced activation of Cdc42. This would be an important issue to be further addressed in future.

4. In the byr1DDras1G17V background deleting the gpa1 reduces the ability to form elongated cells (compare Fig.7B and 2B). How do authors explain this difference in the context of the signaling network?

Thank you very much for highlighting this important issue. We postulate that activated Gpa1, together with Ste4, generates a signalling platform where PS molecules, including Fus1, assemble. As Fus1 generates condensates that facilitate effective actin polymerisation (Billault-Chaumartin et al., 2022), it subsequently leads to effective shmoo formation. A lack of Gpa1 would severely impair assembly formation. Testing this hypothesis is an important task in future.

5. The micrographs of Spk1-GFP in main figures (e.g. Fig.1, 2, 6,7) is far worse quality that that in the Fig.S4, which produces more convincing images. It is possibly due to the quality of the figures I received. In either case, the quality should be improved and quantifications of the nucleo-cytoplasmic ratio should be provided where relevant.

We appreciate the comments. The micrographs in question were taken with a widefield microscope with the intention of obtaining cell morphology information via brightfield images, rather than cellular GFP signal localisation. However, we fully agree with the reviewer that it is important to obtain higher-quality Spk1-GFP images. We therefore reproduced these micrographs using a confocal microscope and replaced the old images with those with improved Spk1-GFP signals.

We conducted quantification of the Spk1-GFP signal in wildtype cells at various stages of the sexual differentiation process. These cells were induced for sexual differentiation for at least 6 hours, so that the GFP signal would be readily detectable and could be used to define the "total cell volume", comprising of cytoplasmic and nuclear areas. To define the area of the nucleus, tdTomato-tagged Smd2 (small nuclear ribonucleoprotein) was used. We obtained higher-resolution images using an Airyscan microscope, and 3D image segmentation was performed using the Spk1-GFP and Smd2-tdTomato signals by Imaris software. We then measured the Spk1-GFP signal in the two segments, the total and the nucleus.

The result demonstrated that both total and nuclear Spk1-GFP levels peaked at the time of mating and karyogamy and declined thereafter. The nuclear-to-total ratio remained constant during the peak period, but later, as the total GFP level declined, the nuclear portion increased slightly. The result is presented in the Supplementary Figure S4.

Having seen an accumulation of the Spk1-GFP at the conjugation tip in the byr1-DD mutant (Supplementary Fig. S8), we strongly believe that active Spk1 resides at the plasma membrane, in addition to the nucleus. Therefore, we are not entirely sure whether the nuclear-cytoplasmic ratio of the Spk1-GFP signal provides information on Spk1 activation status. We would like to address the issue by generating a yeast strain that enables segmentation of the cell cortex for localisation of the cell cortex and the nucleus in the future.

6. The model should be explained in much more detail in the materials and methods. This should include reasoning and justifications, including supporting publications, for proposed reactions (e.g. k15). Have any of the constants been mesured before, including other yeast or in vitro systems? Would any of such measurments restrict the model given that some k (e.g k14, k15, k6) values vary by orders of magnitude between models?

We appreciate the reviewer's insightful comment. In the following sections, we respond to each point in detail.

First, regarding the request for a more detailed explanation of the model, we have revised the Materials and Methods section to explain each equation in natural language. For each equation, we now explicitly describe the biological meaning of individual terms and, where applicable, provide supporting publications. We believe that these additions fully address the reviewer's request for clearer reasoning and justification of the proposed reactions.

Second, regarding whether any of the model parameters have been experimentally measured, the answer is no. To our knowledge, none of the parameters used in the present model has been directly measured in fission yeast, nor is such a measurement straightforward. This is because the parameters in our model do not correspond to single elementary biochemical reactions. Instead, they represent effective reaction efficiencies of coarse-grained nodes (such as the Byr1 or Cdc42 nodes), each of which summarises multiple molecular processes. As such, these parameters cannot be obtained by measuring individual enzymatic reactions *in vitro*. We clarified this point in the revised manuscript as follows (L434): Because many kinetic parameters of the pheromone signaling pathway have not been experimentally determined in fission yeast, model parameters were treated as effective quantities representing coarse-grained regulatory influences rather than elementary biochemical reaction rates, and the reported values therefore represent best-fit examples rather than unique or physiologically exact intracellular parameters.

Importantly, our analysis is designed to focus on what can be concluded even under the assumption that the true intracellular parameter values are difficult or impossible to determine. The purpose of the mathematical modeling in this study is therefore not to infer biologically exact parameter values, but to compare alternative candidate models. We clarified this point in the revised manuscript as follows (L441): Importantly, the purpose of the modeling in this study is not to infer biologically exact kinetic parameter values, but to evaluate alternative candidate network structures based on their ability to account for this characteristic transient $MAPK^{Spk1}$ response, as well as the experimentally measured temporal dynamics of total Spk1 and active Cdc42.

Specifically, we compared Models A, B, and C by systematically searching for best-fit parameter sets that could reproduce the quantitative time-course data of $MAPK^{Spk1}$ phosphorylation and active Cdc42. Under this approach, Model A failed to reproduce the experimentally observed transient increase and subsequent decrease of Spk1 phosphorylation for any parameter set tested, whereas Model B successfully reproduced the data. Because Model B differs from Model A only by the introduction of a delayed negative feedback, this result supports the existence of such a feedback at the network level. In this analysis, the key question is whether parameter sets that explain the data exist or not, rather than the specific numerical values of those parameters. We clarified this point in the revised manuscript as follows (L463): Notably, whilst Model A failed to reproduce the transient increase and subsequent attenuation of $MAPK^{Spk1}$ for any parameter set tested, Model B successfully reproduced these dynamics, indicating that the difference between the models is structural rather than parametric.

We note that the parameter values reported in the manuscript represent best-fit examples. We do not exclude the possibility that other parameter sets may also reproduce the experimental dynamics to a reasonable extent. Consequently, the conclusions of this study do not depend on the assumption that the reported best-fit parameters correspond to the true intracellular values.

Finally, we emphasize that the primary constraints on our model are not unmeasured kinetic parameters, but the experimentally quantified time-course data of total and phosphorylated $MAPK^{Spk1}$ and active Cdc42 in wild-type and mutant strains. In this sense, the modeling presented here is not detached from experimental measurements, but rather constitutes a data-driven analysis grounded in quantitative experimental observations.

7. Quantifications of the tSpk1 should accompany those of ppSpk1. This is particulary important in instances where the mutants severely downregulate tSpk1(Fig.3D,E ; 6A,C), which should be highlighted and explained in the text also.

Thank you very much for raising this important issue, which was neither clearly stated nor discussed in the original manuscript. We provided the total Spk1 quantification in the revised Supplementary Figures S10 and S14 and explained our observation in the text.

Minor points

1. The manuscript is very dense and difficult to read, and more clarity would be appreciated. The authors present the work around the constitutively active Ras, but they study more globally how fission yeast pheromone signaling pathway functions. This results in initial confusion/misleading of the reader. For example, the reason for MAPK singaling attenuation is due to upstream, Ras1-independent regulation, which becomes clear only late in the manuscript.

Thank you very much for the valuable comment. We agree that this study aimed to obtain a holistic view of fission yeast pheromone signalling to explain how constitutively active Ras exerts distinct influences on two downstream pathways, rather than solely studying constitutively active Ras.

We revised the Abstract and Introduction sections to include information about our approach (the genetic epistasis analysis and biochemical approach), so the reader could better grasp the flow of our work.

2. How do authors explain that overexpression of scd1-PB1 and byr2-RBD has little to no effect on tSpk1 but a strong effect on ppSpk1?

Thank you very much for flagging up this point. Our current interpretation is that the degrees of the ppSpk1 reduction are not as severe as the cases caused by the loss of Byr2 (Fig. 3F) or Byr1 (Supplementary Fig. S1G). In the latter two cases, we could not detect ppSpk1 in the western blotting. The lack of the Byr2-Byr1-Spk1 signalling axis means that the pheromone-induced gene expression is severely compromised, leading to a substantially reduced level of total Spk1.

In contrast, overexpression of scd1-PB1/byr2-RBD caused a reduction in the ppSpk1 level to about a half or a third. Although reduced, the generated active Spk1 could have driven pheromone-induced gene expression, leading to Spk1 protein production.

3. Introduce the axis for the mating in Fig.1C.

Thank you very much for the comment. We revised Fig. 1C according to the suggestion.

4. Axis labels missing in Fig.3D, 3E, 6A, 6C, 7A, 7C.

Thank you very much for the comment. We labelled the Y-axis of these graphs.

5. Why is the profile of ppSpk1/tSpk1 different in Fig5A(right pannel, pREP1) and Fig.S3C (ras1G17V)?

We believe that the difference is caused by the media used in the Fig.5 experiment. In this experiment, vegetative growth was maintained in synthetic media (MM+N) rather than YE to ensure that the cells retained the plasmid.

Although we did not let the cells grow beyond 4 × 10^6 cells/ml in the MM+N media, cells may have been expressing PS components at a very low level, ready to produce ppSpk1. This may have led to an increased ppSpk1/total Spk1 ratio, especially when the level of total Spk1 was very low (at time 0).

6. A better definition and qunatification of the "fus" vs. "elongated" phenotypes would be beneficial.

Thank you very much for the comment. We revised the term "fus" to "paired" to highlight the fact that cells could find a mating partner, but fail to fuse.

7. Fig. S12/s14 say Model B in graphs, but presumably it's models A/C.

Thank you very much for pointing this out. We have revised the typo.

Reviewer #2: Reviewer Comments to the Authors:

This manuscript by Kelsall et al. investigates two key aspects of the yeast mating pathway: the pheromone-induced MAPK signalling cascade and the morphological changes leading to shmoo formation and cell-cell pairing. Although the major components of this pathway and their roles in mating are well characterized, and some of the findings reported here overlap with previous studies, the authors employ a valuable approach by analyzing synchronous

mating cultures and tracking MAPK pathway activation over time. Specifically, they monitor the phosphorylation status of the MAPK Spk1 via western blot analysis.

The central finding, supported by mathematical modeling, is that constitutive activation of Ras1, a small GTPase positioned upstream of both the MAPK cascade and the Cdc42-mediated morphological response, does not hyperactivate the pheromone response. Instead, it primarily affects Cdc42 activation, thereby promoting the formation of elongated shmoos.

Overall, I consider the manuscript a meaningful contribution to the field. It offers new insights into a well-characterized signalling pathway through a novel experimental design. I support publication in PLOS Genetics pending revisions. My specific comments are provided below:

- "Gpa1, the α-subunit of the pheromone receptor-coupled G-protein, relays the pheromone signal into the cell through Ras1, leading to the activation of the MAPK cascade" I recommend narrowing this statement. While it is known that the Ras1-GEF Ste6 is activated downstream of Gpa1 and the pheromone–MAPK signalling, MAPK activation also involves inputs that are independent of Ras1.

- Figure 1C: Regarding WT mating efficiency over time - does the percentage refer to mating pairs or zygotes? Please clarify what is represented. Also, the y-axis on the left is labeled as A.U. (arbitrary units), to quantify the pSpk1 signal. Can you specify what the percentage is?

Thank you very much for pointing this out. We have revised the figure to clarify the status of mating and sexual differentiation in the cell population. We counted zygotes as two cells to reflect how the original population of the cells transitioned from a single cell to a zygote. We revised the Fig.1 legend to clarify this point as follows (L1386).

For the wildtype samples in (C), the % ratios of cells at the indicated sexual differentiation stages are also indicated. In this counting, each zygote was counted as two cells to reflect the mating status of the whole population (n=400, three biological replicates).

- Synchronous mating assay: How does the mating efficiency in your assay compare to previously established methods for synchronous mating?

Conventional mating assays involve transferring a mass of cells to solid media lacking nitrogen. These assays are good to test sterile phenotypes. However, for reproducible time-course experiments, they are inappropriate, as the number of cells and the area of the cell spread are not rigorously defined (the mating efficacy is dependent on the cell density – too low is obviously bad, but too high is no good, either). Furthermore, the carry-on nitrogen source from the vegetative culture introduces further complications; if cells are not "washed", residual nitrogen will inhibit the sexual differentiation process. If cells are washed through a couple of centrifugations, they undergo a stress response, which perturbs pheromone signalling.

Cell mating can also be induced in liquid culture. In this case, however, mating begins when the cells are clumped together. Therefore, the way the medium swirls in the flask matters, meaning that not only cell density, but also flask shape, culture volume, and shaker speed all affect mating efficacy. Even if they are accurately defined, each cell clump varies in size, generating heterogeneous mating.

The protocol we describe here addresses these issues to achieve highly reproducible synchronous sexual differentiation, enabling us to conduct time-course analysis of various strains.

- "Signal was first detected three hours after induction of mating and reached its peak at about five to seven hours, when cell fusion was also initially observed" How is cell fusion monitored in your assay? Please specify the method used.

Cell fusion was monitored by cell morphology. Based on brightfield images from multiple Z-stacks, we determined the timing of cell fusion.

- Regarding western blots in supplementary figures, the authors use actin as a loading control in some blots and tubulin in others. Is there a specific reason for switching between

these controls? Additionally, in some cases the loading controls appear weak or inconsistent, making interpretation of the blots less straightforward, even though the quantification presented in the graphs is relatively clear.

Thank you very much for asking about the quality of the Western blotting, which is essential for this study.

Proteomics studies showed that during sexual differentiation, both actin and alpha-tubulin levels remain constant until sporulation begins (Krapp et al., 2019, PMID: 30673600). During the sporulation process, actin remains constant, whereas the level of a-tubulin decreases. Therefore, we used actin as an internal control when Western blotting involved wild-type cells that undergo sporulation. We also used a-tubulin as an internal control when comparing sporulation-deficient mutant strains. The benefit of alpha-tubulin was that the signal intensity was more vigorous, enabling more sensitive quantitation.

We conducted three biological replicates and used a lysis buffer containing urea to minimise inconsistencies in protein solubilisation. Hence, overall, the Western blotting results are reliable. We agree that some of the signals appear weaker than others in the Western blot images, but they were still good to quantify using the Odyssey scanner.

- Although you measure Spk1 levels and phosphorylation via western blot, it would be informative to quantify Spk1-GFP levels from microscopy images, at least in WT, ras1-G17V, and byr1-DD cells. For instance, based on the images, Spk1-GFP expression does not appear to decrease significantly in ras1-G17V cells. Have you considered quantifying the nuclear-to-cytoplasmic ratio of the signal? This could provide additional insights into Spk1 localization and activation.

We greatly appreciate the suggestion. We fully agree that quantifying Spk1-GFP levels by imaging is highly informative and that image analysis can be a powerful strategy to confirm Western blotting results.

Following the reviewer's suggestion, we conducted detailed image analysis of GFP-Spk1 in wildtype cells undergoing sexual differentiation by Airyscan imaging for an improved X-Y-Z resolution, as mentioned earlier in response to Reviewer 1 and presented the outcome in Supplementary Fig. S4. The result confirmed the plasma membrane and nuclear localisation

of GFP-Spk1. Furthermore, the GFP-Spk1 level peaks at the time of cell fusion, confirming the Western blot result for total Spk1.

We also agree that some *ras1.G17V* cells appear to retain a higher level of GFP-Spk1 than wildtype cells in the presented images. So, we tried to obtain cell images that represent the whole population of *ras1.G17V* samples. However, this turned out to be a challenging task, as *ras1.G17* cells form clumps, containing a morphologically highly heterogeneous cell population. When we gently squash cell clumps to let them lie as a single layer for imaging, many small cells escape and are washed away in the flow, leaving a heterogeneous situation of clumped cells and free cells, which often show a low GFP-Spk1 signal. A solution could be to use a light-sheet microscope to image the cell clump as a whole, then segment individual cells by image analysis. Although it would be of great interest to examine the *ras1.G17V* population in detail using imaging to estimate the bulk population status, we would like to leave this as a future task.

- CRIB-GFP signal in ras1Δ cells: The statement, "During the vegetative cycle, ras1Δ cells show spherical morphology where polarised localisation of active Cdc42 is compromised," is accurate but was also previously demonstrated and quantified in Lamas et al., 2020 using CRIB-GFP localization at the cell ends.

Thank you very much for the helpful comment. We cited the reference (L226).

- Some of the fluorescence microscopy images appear out of focus or blurry. Would it be possible to replace these with higher-quality images to improve data presentation?

Thank you very much for the helpful comment. We have replaced the images obtained with a widefield microscope with those obtained with a confocal microscope to improve image quality.

- Figure 3: Please include quantification of the pSpk1 signal in scd1Δ ras1-G17V and scd1Δ byr1-DD strains.

Thank you very much for the suggestion. We included the data in the revised Figure 3D and Supplementary Figure S10A and D.

- Have you examined the effects of expressing byr2-RBD or scd1-PB1 during mitosis? Are there any morphological phenotypes associated with their expression at this stage?

Thank you very much for asking. Expressing byr2-RBD or scd1-PB1 induces morphological change during mitosis. Example images are included in Supplementary Fig. S12.

- Line 238: This should read rga4, not rgl4.

Thank you for the comment. It is now corrected.

- Line 241: While the point is valid, it has been previously shown that Ras activity, together with scaffold-mediated positive feedback, promotes Cdc42 activation.

Thank you very much for pointing this out. We have revised the sentence and cited the relevant work from the Ladds lab (Weston et al., 2013) (Line 289).

- Figure S12: The label “Model B” in the graph should be corrected to “Model A.”

Thank you very much for flagging this up. We have revised the typo.

- Line 438: The correct notation should be Ras1-G17V.

Thank you for the comment. It is now corrected.
