# Supplementary material for: Constitutively active RAS prolongs Cdc42 signalling, while MAPK signalling is attenuated during fission yeast mating
Source: PLoS Genet. 2026 Apr 17;22(4):e1012117. doi: 10.1371/journal.pgen.1012117 (PMC13108886; doi:10.1371/journal.pgen.1012117)
Supplement: Attachment — Submitted filename: Response to the reviewers_March2026.pdf [file pgen.1012117.s027.pdf]

Response to the Reviewers

We thank all the reviewers for their valuable and constructive comments and suggestions. Specific responses to each comment are provided below.

Reviewer 1

I thank the authors for their efforts in addressing most of the points from the initial review. Thus, I would like to recommend publishing the article.

We appreciate Reviewer 1's recognition of the improvements made in the revised manuscript. We are pleased that Reviewer 1 has recommended the article for publication.

However, I would also like to note that it remains unclear how much competition of Byr2 and Scd1 for Ras1-GTP there is at native protein levels (point 2a). The new imaging data suggests "that these two molecules are available at the fusion site at comparable levels". This also suggests that having used the pREP1 vectors (nmt1 protein is 57312.32 molecules/cells) makes it hard to strengthen the idea that Scd1 (453 molecules/cell) and Byr2 (unknown molecules/cell, suggestedly comparable to Scd1) compete for Ras1 (7003.3 molecules/cell for total Ras1 protein, which is order of magnitude greater than either Scd1 or Byr2). For this reason, I would like to recommend to further tone down their conclusions and state explicitly that the extent of Scd1 Byr2 competition in vivo remains to be seen. This is not to take away from the solid biochemistry work in the current manuscript; quite to the contrary, it is to encourage the difficult but important future work on addressing the Scd1 Byr2 competition in vivo.

Thank you very much for highlighting the critical point that the number of Ras1 molecules *in vivo* may exceed that of Scd1 and Byr2 by more than 10-fold. If this is the case, and most Ras1 molecules are in their active state, the competition between Scd1 and Byr2 may not occur for the abundant active Ras1 molecules. We fully agree with Reviewer 1 that further investigation, such as counting the number of molecules at the cell fusion site for active Ras1, Scd1 and Byr2, is required to draw a conclusion about the *in vivo* competition status. Therefore, we have revised the text to emphasise that we cannot make a hasty conclusion and future *in vivo* work is essential for the competition matter as follows.

L312-L314

Collectively, two Ras1 effectors, MAPKKK^{Byr2} and Cdc42-GEF^{Scd1}, are capable of competing with each other for Ras1 *in vivo* when overexpressed.

L332-333

hence, a competition between MAPKKK^{Byr2} and Cdc42-GEF^{Scd1} might exist, provided that the number of available Ras1 molecules is limited.

L582-587

However, this observation was obtained under effector overexpression conditions, in which the abundance of effector molecules likely exceeded that of active Ras1. Future studies should examine the endogenous levels of active Ras1, Scd1 and Byr2 at the fusing cell tips to determine whether effector competition contributes to the regulation of the fission yeast RAS signalling.

L1537

The diagram highlights the bipartite mechanism to activate MAPKKK^{Byr2}, the branch for two Ras1 effectors, MAPKKK^{Byr2} and Cdc42GEF^{Scd1}, and the interplay between the MAPK^{Spk1} and Cdc42 pathways.

Reviewer 2

The reviewer is satisfied with the authors' revisions to the text and figures and supports the manuscript for publication.

We are pleased to hear that our revision has appropriately addressed the raised concerns, and Reviewer 2 has recommended the article for publication.
